# Association between internet addiction and sleep quality in medical students: a longitudinal study

**DOI:** 10.3389/fpsyg.2025.1517590

**Published:** 2025-03-12

**Authors:** Chaowei Guo, Ming Chen, Xiaotong Ji, Jiang Li, Yi Ma, Shuang Zang

**Affiliations:** ^1^Department of Community Nursing, School of Nursing, China Medical University, Shenyang, China; ^2^Teaching and Research Department of P.E., China Medical University, Shenyang, China; ^3^Student Affairs Office, School of Nursing, China Medical University, Shenyang, China; ^4^Department of Otolaryngology, The First Affiliated Hospital of China Medical University, Shenyang, China

**Keywords:** internet addiction, sleep quality, medical students, long-term impact, cross-lagged panel model

## Abstract

**Objective:**

The study aimed to confirm the hysteresis effect of internet addiction on sleep quality and examine the association between internet addiction and sleep quality among medical students from the first to the third academic year.

**Methods:**

A repeated measures observational cohort study was conducted, involving 667 medical students at China Medical University from 2017 to 2019. The Kruskal-Wallis test was used to analyze repeated measurement data, and cross-lagged panel models were employed to assess the associations between internet addiction and sleep quality within and across different time intervals.

**Results:**

Internet addiction was significantly associated with sleep quality (*p* < 0.001). Notably, internet addiction in the first year was positively associated with sleep quality in the second year.

**Conclusion:**

This study underscores the importance of understanding the association between internet addiction and sleep quality as medical students progress through their academic years. Attention should be directed towards the long-term adverse effects of internet addiction on the future sleep quality of medical students.

## 1 Introduction

According to the 53rd Statistical Report on the Development of the Internet in China, as of December 2023, the number of internet users in China reached 1.092 billion, reflecting an increase of 24.80 million from December 2022, and an internet availability rate of 77.50%. The rapid growth of internet usage has been linked to a rise in internet addiction (Javaeed et al., 2020). Research has demonstrated that internet addiction is prevalent among medical students, particularly when the internet is used more for entertainment than educational purposes (Latifeh et al., 2022; Nikolic et al., 2023). Several factors have been identified as contributing to this phenomenon. The most important significant determinants among medical students were unmonitored internet access (Ibrahim et al., 2022). Medical students have greater autonomy and free time, easy access to the internet, and often lack self-control (Adhikari et al., 2022; Rao et al., 2023), so they are more likely to be addicted to the internet. Furthermore, given the internet’s role as a vast network facilitating communication among medical students, social media use, and participation in online activities, there is an increased risk of students spending more time online than intended, thus being labeled as addicted (Masters et al., 2021; El-Zoghby et al., 2024).

A study on medical students in Saudi Arabia suggests that internet addiction is associated with poorer sleep quality (Hammad et al., 2024). Study has shown that approximately 81.62% of medical students suffering from internet addiction experience poor sleep quality (Mahmoud et al., 2022). Previous research has revealed that medical students with internet addiction experience poorer sleep quality than their peers without internet addiction (Kashfi et al., 2023). Maintaining good sleep quality is one of the most critical aspects of medical students’ well-being. However, poor sleep quality is widespread among medical students (Rao et al., 2020). Shafiee et al. (2024a) reported that nearly half of all medical students experience poor sleep quality. The prevalence of poor sleep quality is twice as high among medical students compared to the general population (Wondie et al., 2021). Previous research conducted in countries such as India and Iran has shown that the prevalence of poor sleep quality among medical students ranges from 48.30 to 71.10% (Goel et al., 2023; Shafiee et al., 2024b). Therefore, the sleep quality of medical students should be given due consideration.

Previous study has indicated that internet addiction is associated with a diminished health-related quality of life (Bezgin et al., 2024; Li et al., 2024). Adolescents with internet addiction often immerse themselves in the virtual world for prolonged periods, leading to a reduced perception of health-related quality of life and triggering various related issues (Junior et al., 2024). The decline in health-related quality of life adversely affects the maintenance of good sleep quality (Guclu et al., 2024). Notably, the impact of internet addiction on health-related quality of life is closely linked to morning and night type, further exacerbating sleep quality issues (Lu et al., 2023; Altay and Yavuz, 2024). Morning and night types are primarily manifested as early awakening, daytime drowsiness, and bedtime procrastination (Shakya et al., 2023; Krishnan and Chew, 2024). In this context, physical exercise, as a crucial health intervention, is considered to play a significant moderating role in the relationship between internet addiction and sleep quality (Zhu et al., 2024). Internet addiction leads collegiate students to neglect outdoor activities and physical exercise, making them prone to feelings of excessive energy or restlessness, which in turn hinders their ability to fall asleep (Singla et al., 2023). A close relationship exists between physical exercise and physical fitness. Regular physical exercise can directly improve an individual’s physical fitness. Therefore, increased internet usage can negatively affect an individual’s physical fitness and contribute to sleep-related issues (Duran and Alemdar, 2023).

Internet addiction has disrupted daily activities, particularly among students, leading to neglect of assignments and coursework (Salpynov et al., 2024). The medical profession differs from other disciplines in that medical students not only need to master a vast amount of theoretical knowledge but also undergo clinical training (Angadi et al., 2019). The learning process for medical students is typically accompanied by prolonged periods of high-intensity work and significant emotional stress (Ye et al., 2020; Houri et al., 2023). Social media, online games, and entertainment content have become common outlets for many medical students to relieve stress (Javaeed et al., 2019; Ibrahim et al., 2022). Prolonged immersion in the internet has led medical students to become addicted, resulting in poorer sleep quality and a higher incidence of internet addiction compared to the general population (Zhang et al., 2018; Chauhan et al., 2022). The study of internet addiction and sleep quality among medical students holds significant practical implications. In recent years, the growing issues of internet addiction and sleep quality among medical students have attracted widespread attention, highlighting the need to understand these challenges. Researchers have begun to explore how internet addiction affects medical students’ sleep quality and further analyze its potential impact on their academic performance and career prospects. This research direction not only helps address the current health challenges faced by medical students, but also provides new insights and solutions for the future development of medical education and the mental health support of medical students.

Research suggests that the blue light emitted by mobile phones and other electronic devices can reduce melatonin secretion, which may stimulate the nervous system and impair sleep quality (Lu et al., 2018). Furthermore, the daily decline in sleep quality among individuals with internet addiction may lead to brain damage, potentially exacerbating the severity of internet addiction over time (Demirci et al., 2023). Therefore, long-term internet use, along with reduced melatonin secretion and brain damage, may impair future sleep quality (Sletten et al., 2018). Most current studies on the association between internet addiction and sleep quality of medical students are based on cross-sectional data or one-time assessments, and the evidence does not support conclusions about the longitudinal or hysteresis association between internet addiction and sleep quality. The clear-cut long-term impact of internet addiction on sleep quality is understudied (Tokiya et al., 2020). Given these considerations, we explored whether internet addiction among medical students was associated with sleep quality over the following years of college, hypothesizing that internet addiction and sleep quality would be associated over time. According to the summation effect, when a stimulus is applied to an individual, its intensity increases over time. Therefore, this study conducted a longitudinal investigation to explore the role of internet addiction in the trajectory of sleep quality among medical students during college.

## 2 Methods

### 2.1 Participants and recruitment

This was a longitudinal study that took place from October 2017 to October 2019 in Shenyang City, Liaoning Province. The sampling method in this study was cluster sampling. During the period of study, 21 majors were randomly chosen from a university of Shenyang using a simple random sampling method. A total of 1,265 freshman medical students of the 2017 academic year at China Medical University were randomly selected and evaluated. We analyzed sleep quality, physical fitness, health-related quality of life, morning and night types, and internet addiction among medical students, following them annually for 3 years.

### 2.2 Eligibility criteria

The inclusion criteria were freshman medical students of the 2017 academic year from China Medical University. Those medical students who did not participate consecutively in the studies conducted in 2017, 2018, and 2019 were excluded. Moreover, medical students who are interning at hospitals were excluded.

### 2.3 Data gathering

We used the Questionnaire Star platform to conduct a questionnaire survey of medical students university in Shenyang, China. The questionnaire collected data on sex, age, major, sleep quality, internet addiction, morning and night types, health related quality of life, and physical fitness, using various rating scales. Sleep quality was measured using the Pittsburgh Sleep Quality Index (PSQI) (Liu et al., 1996), internet addiction using the Internet Addiction Test (IAT) (Young, 1998), morning and night types using the Morningness-Eveningness Questionnaire (MEQ) (Horne and Ostberg, 1976), health related quality of life using the Health-Promoting Lifestyle Profile II (HPLP-II) (Walker et al., 1987), and physical fitness using the Chinese National Student Physical Fitness Standard.

The researchers distributed the survey questionnaires through the Questionnaire Star platform, utilizing an online survey format. For medical students who faced difficulties completing the survey, interviewers could offer assistance, but the assistance was provided without leading questions. Explanations were given for any unclear points, ensuring consistency and rationality in the interpretation of each item. The researchers reviewed each completed questionnaire to confirm its completeness, discarding those that were incomplete (e.g., responses with systematic patterns or missing more than 10% of the items). Data analysis was conducted with guidance from statistical experts to ensure objectivity and scientific rigor.

### 2.4 Ethical issue

The protocol of this study was approved by the Ethics Committees of China Medical University (No. CMU12104000).

### 2.5 Sample size

Sample size was estimated using G-power software (3.1.9.7). Based on a previous study in which R^2^ was reported to be 0.06 (Tan et al., 2016). 206 subjects were needed after estimating the condition of a type I error 0.05 to a power of 0.95. In 2017, 1,265 participants were recruited to account for attrition and unknown sources of error.

### 2.6 Outcome measures

The PSQI was used to assess the sleep quality of medical students over the past month (Liu et al., 1996). The scale includes seven component scores (ranges 0–3): (i) subjective sleep quality (very good to very bad), (ii) sleep latency (≤15 min to >60 min), (iii) sleep duration (≥7 h to <5 h), (iv) sleep efficiency (≥85 to <65% hours sleep/h in bed), (v) sleep disturbances (not during the past month to ≥3 times per week), (vi) use of sleeping medications (none to ≥3 times a week), and (vii) daytime dysfunction (not a problem to a very big problem). Each dimension is scored from 0 to 3, with the total score being the sum of the seven dimensions (ranging from 0 to 21). A PSQI global score higher than 5 indicates poor sleep quality (Buysse 3rd et al., 1989). A higher score indicates poorer sleep quality.

The IAT was used to assess the internet addiction and the severity of addiction of medical students (Young, 1998). The IAT comprises 20 items across six dimensions of internet use: psychological dependence, compulsive use, internet withdrawal, problems at school or work, productivity at home, and time management. Responses are measured on a five-point Likert scale ranging from 1 “rarely” to 5 “always” or “does not apply.” A score above 50 indicates internet addiction, with higher scores reflecting greater levels of addiction. In this study, the scale’s Cronbach’s alpha was 0.894, indicating high reliability.

Morning and night types were tested using the MEQ (Horne and Ostberg, 1976). The MEQ consists of 19 questions regarding preferred sleep time and daily performance. Scores range from 16 to 86, categorizing students into three groups: morningness (getting up early and going to bed early, scores 59–86), intermediate (regular type, scores 42–58), and eveningness (late sleep schedules and late wake-up, scores 16–41).

The health related quality of life among medical students was assessed by HPLP-II (Walker et al., 1987). The HPLP-II comprises 52 items across six dimensions: health responsibility, physical activity, nutrition, spiritual growth, interpersonal relations, and stress management. Responses are provided on a four-point Likert scale ranging from 1 “never” to 4 “usual.” The total HPLP-II score ranges from 52 to 208, with scores of 52–104 indicates poor health, 105–156 indicating moderate health, and 157–208 indicating good health. Higher scores reflect a healthier lifestyle. In our study, the Cronbach’s alpha of the scale was 0.959, indicating excellent reliability.

The Chinese National Student Physical Fitness Standard was used to assess physical fitness (Ministry of Education of the People’s Republic of China, 2014). The total score ranges from 0 to 120, with higher scores indicating better physical health. The physical fitness tests included body mass index (BMI), vital capacity, standing long jump, 50-meter run, sit-up (for girls) or pull-up (for boys), and 800-meter (for girls) or 1,000-meter (for boys) run. The testers, who participated in Liaoning province student physical fitness health standard test and received one to three training sessions, formed the test group to test students’ physical fitness. Testing procedures and methods followed the standard guidelines set by the Ministry of Education of China for physical fitness evaluation.

### 2.7 Statistical analysis

Based on the sample size, the Kolmogorov–Smirnov test was used to assess the normality of the data (Massey, 1951). The normality test revealed a skewness value of 0.888 and a kurtosis value of 1.495, indicating that the data were not normally distributed. Consequently, the median and interquartile range (IQR) were computed for continuous variables, and proportions were analyzed for categorical variables. The Kruskal-Wallis test was applied to compare non-normally distributed data across multiple grades. Generalized estimating equation analyses were conducted to assess the association of various factors with sleep quality. Additionally, cross-lagged path analysis was used to examine the temporal association between changes in sleep quality and changes in internet addiction. All analyses were performed using IBM SPSS (IBM SPSS Statistics for Windows, Version 23.0) and Mplus (Version 7.4), with statistical significance set at a two-tailed *p*-value of <0.05.

### 2.8 Sensitivity analysis

We explored the potential for unmeasured confounding between internet addiction and sleep quality by calculating E-values (Haneuse et al., 2019). The E-value quantifies the required magnitude of an unmeasured confounder that could negate the observed association internet addiction and sleep quality.

## 3 Results

**Figure 1:**
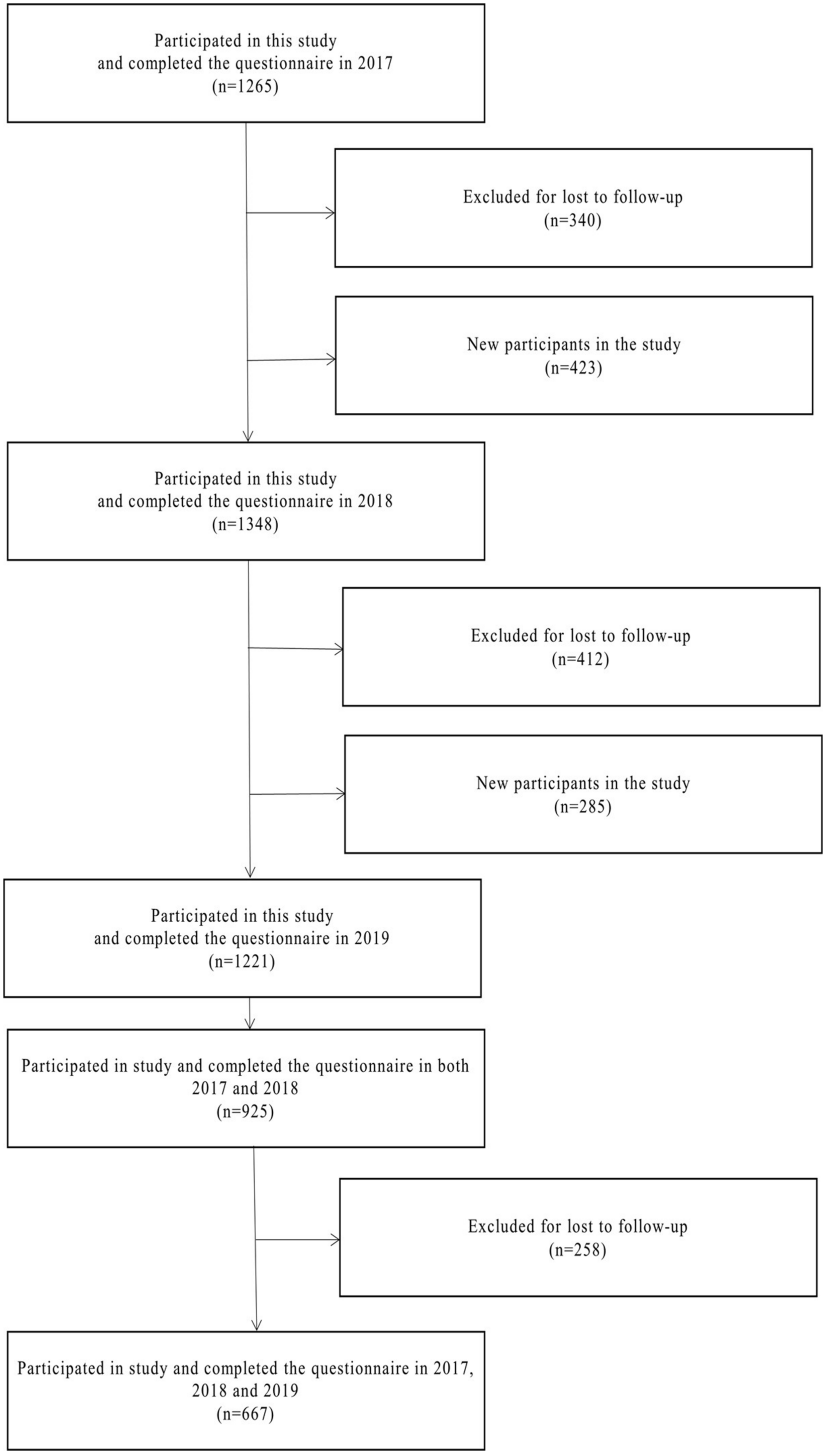
Flowchart of sample selection.

In 2017, a total of 1,265 medical students participated in this study. During the follow-up, 925 students (73.12%) attended the survey in grade 2 (2018). Of the 925 students, 667 (52.73%) participated in the study during grade 3 (year 2019) (52.73%). For more details, see Figure 1. Among the participants in 2017, 62.40% were female and 37.60% were male. The majority of students (59.10%) were aged 22, 23.40% were 21, and 2.20% were aged 24 years or older. This study included students from 21 different majors, with clinical medicine representing the largest group (41.40%). The values for sleep quality, physical fitness, health-related quality of life, morning and night types, and internet addiction are expressed as median (IQR). The study variables, including sex, age, major, physical fitness, health-related quality of life, morning and night types, sleep quality, and internet addiction, are summarized in Table 1.

**Table 1:** Attrition status and summary statistics for critical characteristics by year, N (%), M (IQR).

Sociodemographic indicators	Grade 1 (*N* = 1,265)	Grade 2 (N = 925)	Grade 3 (N = 667)
Sex, *N* (%)			
Male	476(37.60)	319(34.50)	207(31.00)
Female	789(62.40)	606(65.50)	460(69.00)
Age, *N* (%)			
≤17	-	-	-
18	-	-	-
19	2(0.20)	-	-
20	12(0.90)	2(0.20)	-
21	296(23.40)	10(1.10)	1(0.10)
22	747(59.10)	220(23.80)	6(0.90)
23	180(14.20)	544(58.80)	160(24.00)
≥24	28(2.20)	149(16.1)	500(75.00)
Major, *N* (%)			
Clinical medicine	524(41.40)	372(40.22)	253(37.93)
Basic medicine	22(1.70)	18(1.95)	13(1.95)
Preventive medicine	49(3.90)	35(3.78)	28(4.20)
Nursing	106(8.40)	85(9.19)	73(10.94)
Forensic medicine	37(2.90)	29(3.14)	18(2.70)
Medical imaging	42(3.30)	32(3.46)	22(3.30)
Stomatology	38(3.00)	28(3.03)	19(2.85)
Biomedical engineering	50(4.00)	35(3.78)	26(3.90)
Clinical pharmacy	80(6.30)	60(6.49)	44(6.60)
Public utilities management	19(1.50)	8(0.86)	6(0.90)
Information management information system	19(1.50)	17(1.84)	10(1.50)
Pediatrics	37(2.90)	29(3.14)	23(3.45)
Bioscience	40(3.20)	29(3.14)	27(4.05)
Medical laboratory technology	20(1.60)	14(1.51)	10(1.50)
Medical imaging technology	20(1.60)	19(2.05)	12(1.80)
Rehabilitation therapeutics	32(2.50)	25(2.70)	20(3.00)
Anesthesiology	39(3.10)	26(2.81)	22(3.30)
Optometry	16(1.30)	14(1.51)	11(1.65)
Psychiatry	19(1.50)	14(1.51)	8(1.20)
Pharmaceutical preparations	28(2.20)	20(2.16)	11(1.65)
Biotechnology	28(2.20)	16(1.73)	11(1.65)
Physical fitness, median (IQR)	73.40(67.20,78.50)	73.60(66.95,77.90)	71.20(64.20,75.50)
Health related quality of life, median (IQR)	143.00(129.00,159.00)	142.00(128.00,156.00)	144.00(129.00,156.00)
Morning and Night Type, median (IQR)	48.00(45.00,51.00)	48.00(44.00,51.00)	48.00(45.00,51.00)
Sleep Quality, *N* (%)			
Good sleep quality	662(52.33)	487(52.65)	346(51.87)
Poor sleep quality	603(47.67)	438(47.35)	321(48.13)
Internet Addiction, *N* (%)			
Normal	1,112(87.91)	573(61.95)	388(58.17)
Occasional or frequent internet-related problems	153(12.09)	352(38.05)	279(41.83)

* Percentage reported in brackets, which may not add up to 100% due to rounding.

Medical students’ sleep quality significantly decreased in grade 2 compared to grade 1 (*p* < 0.001), but improved in grade 3. However, no statistically significant difference was found between grade 1 and grade 3. Regarding internet addiction, a significant difference was observed across grades (*p* < 0.001). There was a gradual increase in internet addiction as medical students advanced in their grades, with higher levels of addiction in grade 2 compared to grade 1 (*p* < 0.001) (see Table 2).

**Table 2:** Comparing the sleep quality, and internet addiction among three grades, M (IQR).

Indicators	Grade 1 (*n* = 667)	Grade 2 (*n* = 667)	Grade 3 (*n* = 667)	Statistics	*P*-value	Inter group comparison
Sleep quality	5.00 (4.00,7.00)	6.00 (4.00,8.00)	6.00 (4.00,8.00)	29.370	<0.001	S1 < S2 (*P* < 0.001) S2 > S3 (*P* < 0.001)
Internet addiction	35.00 (29.00,43.00)	47.00 (39.00,55.00)	48.00 (40.00,57.00)	349.818	<0.001	I1 < I2 (*P* < 0.001) I1 < I3(*p* < 0.001)

S1 = score of sleep quality in grade 1, S2 = score of sleep quality in grade 2, S3 = score of sleep quality in grade 3; I1 = score of internet addiction in grade 1, I2 = score of internet addiction in grade 2, I3 = score of internet addiction in grade 3.

A generalized estimating equation model was used to examine the association between sleep quality and internet addiction (Table 3). Internet addiction was significantly associated with sleep quality (*p* < 0.001). The findings indicated that medical students with severe internet addiction reported poorer sleep quality.

**Table 3:** Generalized estimating equation analysis of the effect of internet addiction on sleep quality.

Variable	Regression coefficient	SE	$\chi^{2}$ (df)	*p* value
Internet addiction	0.048	0.007	53.47(1)	<0.001
Physical fitness	0.011	0.008	1.67(1)	0.196
Health related quality of life	−0.026	0.003	57.60(1)	<0.001
Morning and night type	0.054	0.015	12.44(1)	<0.001

**Figure 2:**
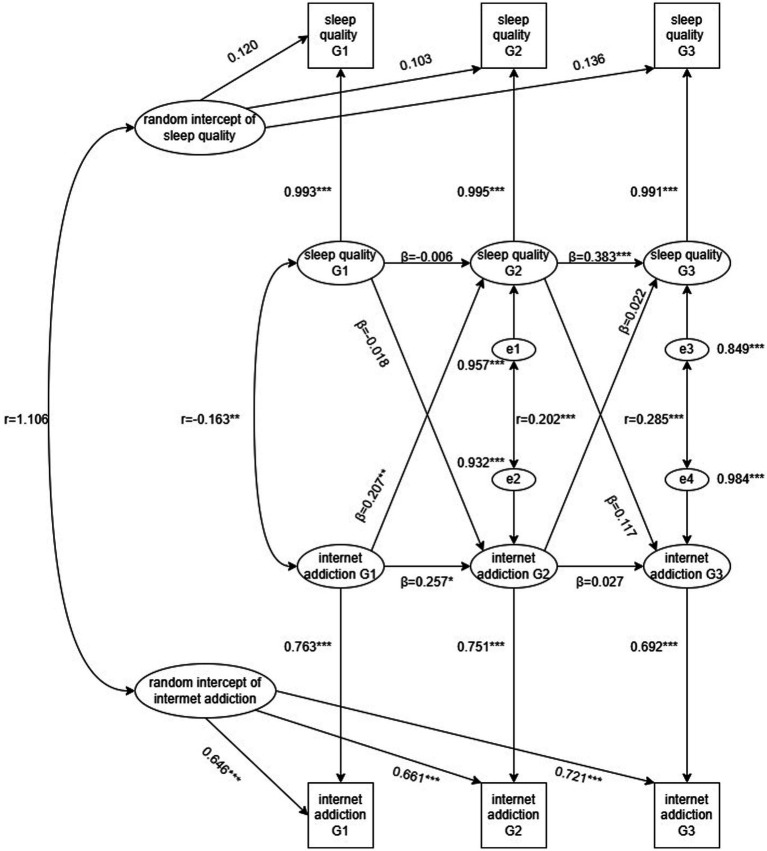
Autoregressive, cross-lagged model for number of sleep quality and internet addiction in grade 1 to grade 3. G1 = grade 1, G2 = grade 2, G3 = grade 3 Partial regression coefficients are on the cross-age paths (*β*). Within-grade correlations are illustrated by dotted lines (r). Due to the inclusion of stability and cross-lagged coefficients in the model, these within-grade correlations function as residuals of sleep quality and internet addiction (e). Residuals reflect associations between sleep quality and internet addiction difference scores that are specific to the grade at which they were measured and independent of any preexisting associations. All paths were retained for regardless of whether coefficients attained significance. *p < 0.05, **p < 0.01, ***p < 0.001.

To understand the extent to which internet addiction can predict sleep quality, we conducted a cross-lagged analysis, with sleep quality as the dependent variable and internet addiction as the predictor variable. The Cross-lagged models included both sleep quality and internet addiction at each time point, with cross-lagged paths illustrating the association between one variable at a previous time point and other at a later time point. The results showed autoregressive relationships for sleep quality and internet addiction over the past 3 years among medical students. The lagged coefficients for internet addiction predicting sleep quality (grade 1 to grade 2 standardized coefficient = 0.207; grade 2 to grade 3 = 0.022) were larger than those for sleep quality predicting internet addiction (grade 1 to grade 2 standardized coefficient = 0.018; grade 2 to grade 3 = 0.117). Internet addiction in grade 1 positively predicted sleep quality in grade 2 (*β* = 0.207, *p* < 0.01). However, Internet addiction in grade 2 did not significantly predict sleep quality in grade 3. Sleep quality scores showed significant positive within-grade correlations between grade 2 and grade 3 (*β* = 0.383, *p* < 0.001), but there was no significant correlation between grade 1 and grade 2. Internet addiction scores showed a significant positive correlations between grade 1 and grade 2 (*β* = 0.257, *p* < 0.05), but no significant correlation between grade 2 and grade 3 (see Figure 2).

### 3.1 Sensitivity analysis

The E-value of this study was 4.39. Based on the E-value, it is unlikely that unmeasured confounding factors could fully explain our results.

## 4 Discussion

This study presents a longitudinal investigation of changes in sleep quality and internet addiction among medical students in their first through third years of university. Based on a longitudinal research design, this study examined the time relationships between sleep quality and internet addiction in a sample of Chinese medical students. To the best of our knowledge, this study is the first to investigate the temporal relationships between sleep quality and internet addiction using a cross-lagged path analysis model, a statistical method for analyzing the causal relationship between interrelated variables. Our findings showed that sleep quality and internet addiction scores declined over the academic years. And within the time point, there were significant associations between sleep quality and internet addiction. The study expands on the lagged impact of internet addiction.

This study reveals that nearly half of the third-year medical students are internet addicts, surpassing the numbers in the first and second years. This finding suggests that the prevalence of internet addiction among medical students increases with higher grades. One potential reason is that senior students, having adapted well to university life, may have more time and energy to spend online (Wang et al., 2020). Additionally, there is a significant correlation between students’ majors and their internet usage (Khazaie et al., 2023), which may stem from the increased necessity for senior medical students to use the internet for study and research. In other words, internet usage varies across different academic years. As students advance, the depth and scope of their learning expand, potentially leading to prolonged internet use (Xu et al., 2020). And senior students may face more demanding academic tasks, which could increase the risk of internet addiction and lead to a decline in sleep quality (Jiang and Yoo, 2024).The analysis indicates a positive correlation between the time spent online and the likelihood of internet addiction (Li et al., 2014). Consequently, senior medical students are at a higher risk of internet addiction. Thus, senior medical students should prioritize effective time management to balance academic responsibilities and leisure activities.

Our findings also indicate that medical students with higher levels of internet addiction tend to experience poorer sleep quality. This is consistent with a study on internet addiction and sleep quality among college students in Taiwan (Cheng et al., 2012). Previous research has shown that medical students with internet addiction often engage in frequent and prolonged internet use, particularly at bedtime, which is positively correlated with poor sleep quality (Chatterjee and Kar, 2021). This may be due to the physical arousal and psychological stimulation caused by internet use before sleep, which can interfere with the ability to fall asleep and negatively affect both sleep duration and quality. Medical students have extremely busy schedules during the academic year, but online games are often difficult to pause. Offline games can be played on various handheld devices, such as smartphones, tablets, and laptops, allowing medical students to enjoy them anytime and anywhere without being restricted by internet access (Alghamdi et al., 2024). A study on university students indicates that internet addiction exhibits an inverted U-shaped relationship with sleep duration (Zhu et al., 2023). This unrestricted internet usage prevents medical students from getting adequate sleep. Therefore, medical students are advised to minimize internet usage before bedtime. Given the demanding nature of their academic workload, it is essential to limit daily internet use to necessary academic activities and appropriate leisure time.

Additionally, the current study found that second-year medical students experienced the worst sleep quality during college. This aligns with Zhou et al. (2022) study on undergraduates’ sleep quality, which concluded that freshmen had better sleep quality than sophomores, who reported poorer sleep quality. This may be because the second year of university is a crucial stage for medical students, where they are typically exposed to more specialized knowledge and clinical courses, increasing their academic burden and affecting their sleep quality (Wang et al., 2022). But some second-year college medical students may not be mature enough in time management, especially when faced with a large number of study tasks, often sacrificing sleep in response to the pressure of their studies (Tran et al., 2023). In contrast to our study, other research on the sleep quality of medical students has yielded different results. For instance, a study of university students found no statistically significant variation in sleep scores between academic years (Saat et al., 2021). Additionally, factors such as academic program type and sleep hygiene are more likely to contribute to sleep quality issues among university students (Ali et al., 2023). However, medical students appear to be more susceptible to sleep quality problems than their peers in other disciplines (Gassara et al., 2016). The discrepancy between our findings and previous studies may stem from different patterns of internet-based entertainment usage among university students, which may vary based on their distinct needs and goals when using the internet. Additionally, the challenges freshman year students face when adjusting to university life are common risk factors for sleep quality problems. Thus, the results of this study align with previous research suggesting that sleep quality issues are multifactorial, rather than solely dependent on major (Ayala et al., 2017).

This study highlights the associations between sleep quality and internet addiction, suggesting that internet addiction among medical students is a significant predictor of sleep quality in subsequent years, emphasizing its long-term adverse impact on sleep during university. For instance, Tahir et al. (2021) reported that internet addiction accounted for 13.2% of the variance in poor sleep quality, indicating its significant predictive role. Additionally, previous research revealed that the effects of internet addiction persist over time (Younes et al., 2016). This may be because, as medical students’ screen time increases, they continuously receive strong motivation and rewards from a sense of control over the internet, instant feedback, and opportunities for self-expression (Zeyrek et al., 2024). Additionally, over time, the intensification of impulsive traits and the worsening of self-control and inhibitory abilities in medical students can contribute to the persistence of internet addiction among them (Kao, 2023). Moreover, once established, medical students’ biological clocks and life rhythm can perpetuate poor sleep habits. Consistent with this, considerable research shows that internet addiction can lead to undesirable outcomes in vulnerable individuals (Buneviciene and Bunevicius, 2021; Alahdal et al., 2023). These findings help explain the lagged effect of internet addiction on sleep quality observed in this study. Therefore, establishing healthy internet use habits early is crucial for ensuring good sleep quality both now and in the future.

This study collected baseline information on various factors, including demographic characteristics, health-related quality of life, morning and night type, and physical fitness. We can control for these baseline variables when analyzing the changes in internet addiction and sleep quality over time. This study calculated the E-value to assess the unmeasured factors that may influence study variables over time, including social life factors (VanderWeele and Ding, 2017). The E-value refers to the minimum strength of association required. A larger E-value indicates that an unmeasured confounder with strong associations are needed to be required to completely negate the current findings, while a smaller E-value suggests that an unmeasured confounder with weaker association are needed to be sufficient to overturn the results. The E-value has been widely used in sensitivity analysis of observational studies (Ding and VanderWeele, 2016; VanderWeele and Ding, 2017; Haneuse et al., 2019). In this study, the E-value indicates that the current findings are robust.

In summary, the longitudinal study showed that there was a dominant cross-lagged effect between internet addiction and sleep quality. Internet addiction was a significant predictor of poor sleep quality. Based on the results of our study, we can propose several key suggestions to mitigate the long-term negative impact of internet addiction on the sleep quality of medical students. Firstly, it is essential to raise awareness about the potential risks of excessive internet use among medical students and encourage them to be more mindful of their screen time. In addition, universities should implement early intervention strategies, such as psychological counseling and guidance to medical students on how to establish regular sleep routines, especially for students who show signs of internet addiction. Universities could also implement regular assessments or surveys to monitor medical students’ internet use patterns and their effects on sleep quality, helping to identify those at risk of developing internet addiction early. This has important implications for prevention of internet addiction and poor sleep quality.

### 4.1 Limitations

This research has certain limitations. Data were collected from a single university, limiting the generalizability of the findings to other populations. Additionally, the reliance on self-reported questionnaires may have introduced biases related to memory and self-evaluation. Although E-value showed robust results in this study, there were still some uncontrollable potential social life factors that influence internet addiction of medical students over time.

## Data Availability

The raw data supporting the conclusions of this article will be made available by the authors, without undue reservation.

## References

[ref1] Adhikari K. Dahal S. Ghimire A. Khanal G. Koirala S. Bhusal C. K.. (2022). Internet addiction and associated factors among undergraduates. J. Nepal Health Res. Counc. 20, 131–137. doi: 10.33314/jnhrc.v20i01.3625, PMID: 35945865

[ref2] Alahdal W. M. Alsaedi A. A. Garrni A. S. Alharbi F. S. (2023). The impact of smartphone addiction on sleep quality among high school students in Makkah, Saudi Arabia. Cureus 15:e40759. doi: 10.7759/cureus.40759, PMID: 37485102 PMC10361753

[ref3] Alghamdi F. A. D.-A. Alghamdi F. A. G. Abusulaiman A. Alsulami A. J. Bamotref M. Alosaimi A.. (2024). Video game addiction and its relationship with sleep quality among medical students. J. Epidemiol. Glob. Health 14, 1122–1129. doi: 10.1007/s44197-024-00265-x, PMID: 38896209 PMC11442900

[ref4] Ali R. M. Zolezzi M. Awaisu A. Eltorki Y. (2023). Sleep quality and sleep hygiene behaviours among university students in Qatar. Int. J. Gen. Med. 16, 2427–2439. doi: 10.2147/IJGM.S402399, PMID: 37333875 PMC10276586

[ref5] Altay G. Yavuz A. Y. (2024). The relationship between chronotype video game addiction and sleep quality in school-age children: a structural equation modeling approach. Chronobiol. Int. 41, 1422–1429. doi: 10.1080/07420528.2024.2419865, PMID: 39445625

[ref6] Angadi N. B. Kavi A. Shetty K. Hashilkar N. K. (2019). Effectiveness of flipped classroom as a teaching-learning method among undergraduate medical students-An interventional study. J. Educ. Health Promot. 8:211. doi: 10.4103/jehp.jehp_163_19, PMID: 31807601 PMC6852382

[ref7] Ayala E. E. Berry R. Winseman J. S. Mason H. R. (2017). A cross-sectional snapshot of sleep quality and quantity among US medical students. Acad. Psychiatry 41, 664–668. doi: 10.1007/s40596-016-0653-5, PMID: 28091977

[ref8] Bezgin S. Özkaya Y. Akbaş Y. Elbasan B. (2024). An investigation of computer-game addiction, physical activity level, quality of life and sleep of children with a sibling with a chronic condition. Child Care Health Dev. 50:e13228. doi: 10.1111/cch.13228, PMID: 38265131

[ref9] Buneviciene I. Bunevicius A. (2021). Prevalence of internet addiction in healthcare professionals: systematic review and meta-analysis. Int. J. Soc. Psychiatry 67, 483–491. doi: 10.1177/0020764020959093, PMID: 32962501

[ref10] Buysse D. J. C F R. Monk T. H. Berman S. R. Kupfer D. J. (1989). The Pittsburgh sleep quality index: a new instrument for psychiatric practice and research. Psychiatry Res. 28, 193–213. doi: 10.1016/0165-1781(89)90047-4, PMID: 2748771

[ref11] Chatterjee S. Kar S. K. (2021). Smartphone addiction and quality of sleep among Indian medical students. Psychiatry 84, 182–191. doi: 10.1080/00332747.2021.1907870, PMID: 33856961

[ref12] Chauhan N. Tiwari P. Ahlawat P. Singh S. K. Kamble B. D. Mahaur G. (2022). Internet addiction and sleep quality among medical students of Delhi: a new age epidemic. Natl. J. Community Med. 13, 864–868. doi: 10.55489/njcm.131220222488

[ref13] Cheng S. H. Shih C.-C. Lee H. Hou Y.-W. Chen K. C. Chen K.-T.. (2012). A study on the sleep quality of incoming university students. Psychiatry Res. 197, 270–274. doi: 10.1016/j.psychres.2011.08.011, PMID: 22342120

[ref14] Demirci E. Tastepe N. Gul M. K. Ozmen S. Kilic E. (2023). S100B and neuron-specific enolase levels as brain injury biomarkers in internet addiction: effect of sleep. Pediatr. Neurol. 149, 93–99. doi: 10.1016/j.pediatrneurol.2023.08.029, PMID: 37837757

[ref15] Ding P. VanderWeele T. J. (2016). Sensitivity analysis without assumptions. Epidemiology 27, 368–377. doi: 10.1097/EDE.0000000000000457, PMID: 26841057 PMC4820664

[ref16] Duran Ş. Alemdar D. K. (2023). Investigation of the correlation between internet addiction, obesity risk and sleep disorder in children. J. Pediatr. Nurs. 73, e409–e417. doi: 10.1016/j.pedn.2023.10.009, PMID: 37863788

[ref17] El-Zoghby S. M. Zaghloul N. M. Tawfik A. M. Elsherbiny N. M. Shehata S. A. Soltan E. M. (2024). Cyberchondria and smartphone addiction: a correlation survey among undergraduate medical students in Egypt. J. Egypt. Public Health Assoc. 99:7. doi: 10.1186/s42506-024-00154-y, PMID: 38565743 PMC10987454

[ref18] Gassara I. Ennaoui R. Halwani N. Turki M. Aloulou J. Amami O. (2016). Sleep quality among medical students. Eur. Psychiatry 33:S594. doi: 10.1016/j.eurpsy.2016.01.2216

[ref19] Goel A. Moinuddin A. Tiwari R. Sethi Y. Suhail M. K. Mohan A.. (2023). Effect of smartphone use on sleep in undergraduate medical students: a cross-sectional study. Health Care 11:2891. doi: 10.3390/healthcare11212891, PMID: 37958035 PMC10649238

[ref20] Guclu Y. Guclu O. A. Demirci H. (2024). Relationships between internet addiction, smartphone addiction, sleep quality, and academic performance among high-school students. Rev. Assoc. Med. Bras. 70:e20230868. doi: 10.1590/1806-9282.20230868, PMID: 38451585 PMC10914330

[ref21] Hammad M. A. Alyami M. H. F. Awed H. S. (2024). The association between internet addiction and sleep quality among medical students in Saudi Arabia. Ann. Med. 56:2307502. doi: 10.1080/07853890.2024.2307502, PMID: 38294763 PMC10833109

[ref22] Haneuse S. VanderWeele T. J. Arterburn D. (2019). Using the E-value to assess the potential effect of unmeasured confounding in observational studies. JAMA 321, 602–603. doi: 10.1001/jama.2018.21554, PMID: 30676631

[ref23] Horne J. A. Ostberg O. (1976). A self-assessment questionnaire to determine morningness-eveningness in human circadian rhythms. Int. J. Chronobiol. 4, 97–110, PMID: 1027738

[ref24] Houri H. N. A. Jomaa S. Arrouk D. M. N. Nassif T. Allah M. J. A. A. Houri A. N. A.. (2023). The prevalence of stress among medical students in Syria and its association with social support: a cross-sectional study. BMC Psychiatry 23:97. doi: 10.1186/s12888-023-04593-3, PMID: 36750821 PMC9906887

[ref25] Ibrahim A. K. Fouad I. Kelly S. J. Fawal B. E. Ahmed G. K. (2022). Prevalence and determinants of internet addiction among medical students and its association with depression. J. Affect. Disord. 314, 94–102. doi: 10.1016/j.jad.2022.07.007, PMID: 35817304

[ref26] Javaeed A. Jeelani R. Gulab S. Ghauri S. K. (2020). Relationship between internet addiction and academic performance of undergraduate medical students of Azad Kashmir. Pak. J. Med. Sci. 36, 229–233. doi: 10.12669/pjms.36.2.1061, PMID: 32063965 PMC6994907

[ref27] Javaeed A. Zafar M. B. Iqbal M. Ghauri S. K. (2019). Correlation between internet addiction, depression, anxiety and stress among undergraduate medical students in Azad Kashmir. Pak. J. Med. Sci. 35, 506–509. doi: 10.12669/pjms.35.2.169, PMID: 31086541 PMC6500801

[ref28] Jiang L. Yoo Y. (2024). Adolescents' short-form video addiction and sleep quality: the mediating role of social anxiety. BMC Psychol. 12:369. doi: 10.1186/s40359-024-01865-9, PMID: 38943173 PMC11214215

[ref29] Junior G. J. F. Silva A. B. D. Meneghetti A. Leite C. R. Brust C. Moreira G. C.. (2024). Relationships between internet addiction, quality of life and sleep problems: a structural equation modeling analysis. J. Pediatr. 100, 283–288. doi: 10.1016/j.jped.2023.09.015, PMID: 38182125 PMC11065653

[ref30] Kao P.-C. (2023). The interrelationship of loneliness, smartphone addiction, sleep quality, and students' attention in English as a foreign language class. Int. J. Environ. Res. Public Health 20:3460. doi: 10.3390/ijerph20043460, PMID: 36834156 PMC9958870

[ref31] Kashfi S. M. Karami H. Jafari F. Daliri M. Yazdankhah M. Kamyab A.. (2023). Internet addiction and sleep disorders among medical students. Sci. World J. 2023:676. doi: 10.1155/2023/6685676, PMID: 37780639 PMC10541298

[ref32] Khazaie H. Lebni J. Y. Abbas J. Mahaki B. Chaboksavar F. Kianipour N.. (2023). Internet addiction status and related factors among medical students: a cross-sectional study in western Iran. Community Health Equity Res. Policy 43, 347–356. doi: 10.1177/0272684X211025438, PMID: 34128427

[ref33] Krishnan A. Chew P. K. H. (2024). Impact of social media addiction and internet gaming disorder on sleep quality: serial mediation analyses. Psychiatry Q. 95, 185–202. doi: 10.1007/s11126-024-10068-9, PMID: 38512552

[ref34] Latifeh Y. Alkhatib Y. Hmidouch M. Swed S. Hafez W. Sawaf B.. (2022). Prevalence of internet addiction among Syrian undergraduate medical students. Medicine 101:e32261. doi: 10.1097/MD.0000000000032261, PMID: 36626507 PMC9750638

[ref35] Li L. Feng X. Luo S. Lin L. Xiang H. Chen D.. (2024). Internet addiction and health-related quality of life in adolescents: the mediating role of sleep disturbance. Sleep Med. 117, 53–59. doi: 10.1016/j.sleep.2024.03.007, PMID: 38507977

[ref36] Li Y. Zhang X. Lu F. Zhang Q. Wang Y. (2014). Internet addiction among elementary and middle school students in China: a nationally representative sample study. Cyberpsychol. Behav. Soc. Netw. 17, 111–116. doi: 10.1089/cyber.2012.0482, PMID: 23971432 PMC3924822

[ref37] Liu X. Tang M. Hu L. Wang A. Wu H. Zhao G.. (1996). Reliability and validity of the Pittsburgh sleep quality index. Chinese J. Psychiatry 29, 103–107.

[ref38] Lu Y. Lin J. Wang F. Liu Y. (2018). Research progress of sleep quality situation of medical students and its influencing factors. World Lates Med. Inf. 18, 35–36. doi: 10.19613/j.cnki.1671-3141.2018.61.018

[ref39] Lu J. Zhai Y. Chen J. Zhang Q. Chen T. Lu C.. (2023). Network analysis of internet addiction and sleep disturbance symptoms. Prog. Neuro Psychopharmacol. Biol. Psychiatry 125:110737. doi: 10.1016/j.pnpbp.2023.110737, PMID: 36868497

[ref40] Mahmoud O. A. A. Hadad S. Sayed T. A. (2022). The association between internet addiction and sleep quality among Sohag university medical students. Middle East Curr. Psychiatry 29:23. doi: 10.1186/s43045-022-00191-3

[ref41] Massey F. J. (1951). The Kolmogorov-Smirnov test for goodness of fit. J. Am. Stat. Assoc. 46, 68–78. doi: 10.1080/01621459.1951.10500769

[ref42] Masters K. Loda T. Tervooren F. Herrmann-Werner A. (2021). How have researchers acknowledged and controlled for academic work activity when measuring medical students' internet addiction? A systematic literature review. Int. J. Environ. Res. Public Health 18:7681. doi: 10.3390/ijerph18147681, PMID: 34300132 PMC8306379

[ref43] Ministry of Education of the People’s Republic of China. _M_ (2014). Notification of National Student Physical Health Standard revised in 2014. Ministry of Education of the People’s Republic of China Available online at: http://www.moe.gov.cn/s78/A17/twys_left/moe_938/moe_792/s3273/201407/t20140708_171692.html (Accessed December 30, 2021).

[ref44] Nikolic A. Bukurov B. Kocic I. Vukovic M. Ladjevic N. Vrhovac M.. (2023). Smartphone addiction, sleep quality, depression, anxiety, and stress among medical students. Front. Public Health 11:1252371. doi: 10.3389/fpubh.2023.1252371, PMID: 37744504 PMC10512032

[ref45] Rao W.-W. Li W. Qi H. Hong L. Chen C. Li C.-Y.. (2020). Sleep quality in medical students: a comprehensive meta-analysis of observational studies. Sleep Breath. 24, 1151–1165. doi: 10.1007/s11325-020-02020-5, PMID: 32072469

[ref46] Rao R. Verma M. Singh C. M. Nirala S. K. Naik B. N. (2023). Internet addiction and nomophobia among medical undergraduates of a tertiary care teaching institute in Patna, eastern India. J. Educ. Health Promot. 12:286. doi: 10.4103/jehp.jehp_1663_22, PMID: 37849868 PMC10578553

[ref47] Saat N. Hanawi S. A. Farah N. M. Amin H. M. Hanafiah H. Selvaraj T. (2021). Associations of physical activity, sleep quality and cardiovascular risk factors in university students. Sustain. For. 13:11806. doi: 10.3390/su132111806

[ref48] Salpynov Z. Kosherova Z. Sarría-Santamera A. Nurkatov Y. Gusmanov A. Semenova Y. (2024). The worldwide prevalence of internet addiction among medical students: a systematic review and Meta-analysis. Int. J. Environ. Res. Public Health 21:1146. doi: 10.3390/ijerph21091146, PMID: 39338027 PMC11430859

[ref49] Shafiee A. Athar M. M. T. Seighali N. Amini M. J. Hajishah H. Bahri R. A.. (2024a). The prevalence of depression, anxiety, and sleep disturbances among medical students and resident physicians in Iran: a systematic review and meta-analysis. PLoS One 19:e0307117. doi: 10.1371/journal.pone.0307117, PMID: 39178292 PMC11343466

[ref50] Shafiee A. Fili J. Ghafari S. Sattari M. A. Borna N. Pourramzani A. (2024b). The prevalence of sleep disturbance and its possible associated factors among iranian medical students: a cross-sectional study with a national meta-analysis. Sleep Med. X 7:100107. doi: 10.1016/j.sleepx.2024.100107, PMID: 38374870 PMC10875233

[ref51] Shakya M. Singh R. Chauhan A. Rure D. Shrivastava A. (2023). Prevalence of internet gaming addiction and its association with sleep quality in medical students. Ind. Psychiatry J. 32, S161–S165. doi: 10.4103/ipj.ipj_236_23, PMID: 38370952 PMC10871426

[ref52] Singla D. Desai O. P. Basista R. Khan S. A. (2023). Association between internet use, sleep, cognition and physical activity levels during COVID-19 lockdown. Sleep Vigil. 23, 1–10. doi: 10.1007/s41782-023-00232-9, PMID: 37361912 PMC10203657

[ref53] Sletten T. L. Magee M. Murray J. M. Gordon C. J. Lovato N. Kennaway D. J.. (2018). Efficacy of melatonin with behavioural sleep-wake scheduling for delayed sleep-wake phase disorder: a double-blind, randomised clinical trial. PLoS Med. 15:e1002587. doi: 10.1371/journal.pmed.1002587, PMID: 29912983 PMC6005466

[ref54] Tahir M. J. Malik N. I. Ullah I. Khan H. R. Perveen S. Ramalho R.. (2021). Internet addiction and sleep quality among medical students during the COVID-19 pandemic: a multinational cross-sectional survey. PLoS One 16:e0259594. doi: 10.1371/journal.pone.0259594, PMID: 34739502 PMC8570473

[ref55] Tan Y. Chen Y. Lu Y. Li L. (2016). Exploring associations between problematic internet use, depressive symptoms and sleep disturbance among southern Chinese adolescents. Int. J. Environ. Res. Public Health 13:313. doi: 10.3390/ijerph13030313, PMID: 26985900 PMC4808976

[ref56] Tokiya M. Itani O. Otsuka Y. Kaneita Y. (2020). Relationship between internet addiction and sleep disturbance in high school students: a cross-sectional study. BMC Pediatr. 20:379. doi: 10.1186/s12887-020-02275-7, PMID: 32782022 PMC7418409

[ref57] Tran D.-S. Nguyen D.-T. Nguyen T.-H. Tran C.-T.-P. Duong-Quy S. Nguyen T.-H. (2023). Stress and sleep quality in medical students: a cross-sectional study from Vietnam. Front. Psych. 14:1297605. doi: 10.3389/fpsyt.2023.1297605, PMID: 38025426 PMC10680167

[ref58] VanderWeele T. J. Ding P. (2017). Sensitivity analysis in observational research: introducing the E-value. Ann. Intern. Med. 167, 268–274. doi: 10.7326/M16-2607, PMID: 28693043

[ref59] Walker S. N. Sechrist K. R. Pender N. J. (1987). The health-promoting lifestyle profile: development and psychometric characteristics. Nurs. Res. 36, 76–81. doi: 10.1097/00006199-198703000-00002, PMID: 3644262

[ref60] Wang Q. Sun W. Wu H. (2022). Associations between academic burnout, resilience and life satisfaction among medical students: a three-wave longitudinal study. BMC Med. Educ. 22:248. doi: 10.1186/s12909-022-03326-6, PMID: 35382810 PMC8980514

[ref61] Wang Y. Zhao Y. Liu L. Chen Y. Ai D. Yao Y.. (2020). The current situation of internet addiction and its impact on sleep quality and self-injury behavior in Chinese medical students. Psychiatry Investig. 17, 237–242. doi: 10.30773/pi.2019.0131, PMID: 32151129 PMC7113173

[ref62] Wondie T. Molla A. Mulat H. Damene W. Bekele M. Madoro D.. (2021). Magnitude and correlates of sleep quality among undergraduate medical students in Ethiopia: cross-sectional study. Sleep Sci. Pract. 5:7. doi: 10.1186/s41606-021-00058-2

[ref63] Xu D.-D. Lok K.-I. Liu H.-Z. Cao X.-L. An F.-R. Hall B. J.. (2020). Internet addiction among adolescents in Macau and mainland China: prevalence, demographics and quality of life. Sci. Rep. 10:16222. doi: 10.1038/s41598-020-73023-1, PMID: 33004842 PMC7529916

[ref64] Ye W. Ye X. Liu Y. Liu Q. Vafaei S. Gao Y.. (2020). Effect of the novel coronavirus pneumonia pandemic on medical students' psychological stress and its influencing factors. Front. Psychol. 11:548506. doi: 10.3389/fpsyg.2020.548506, PMID: 33178063 PMC7591817

[ref65] Younes F. Halawi G. Jabbour H. Osta N. E. Karam L. Hajj A.. (2016). Internet addiction and relationships with insomnia, anxiety, depression, stress and self-esteem in university students: a cross-sectional designed study. PLoS One 11:e0161126. doi: 10.1371/journal.pone.0161126, PMID: 27618306 PMC5019372

[ref66] Young K. S. (1998). Caught in the net: How to recognize the signs of internet addiction-and a winning strategy for recovery. New York: John Wiley & Sons, Inc.

[ref67] Zeyrek I. Tabara M. F. Çakan M. (2024). Exploring the relationship of smartphone addiction on attention deficit, hyperactivity symptoms, and sleep quality among university students: a cross-sectional study. Brain Behav. 14:e70137. doi: 10.1002/brb3.70137, PMID: 39576227 PMC11583478

[ref68] Zhang M. W. B. Lim R. B. C. Lee C. Ho R. C. M. (2018). Prevalence of internet addiction in medical students: a Meta-analysis. Acad. Psychiatry 42, 88–93. doi: 10.1007/s40596-017-0794-1, PMID: 28849574

[ref69] Zhou Y. Bo S. Ruan S. Dai Q. Tian Y. Shi X. (2022). Deteriorated sleep quality and influencing factors among undergraduates in northern Guizhou, China. PeerJ 10:e13833. doi: 10.7717/peerj.13833, PMID: 36039370 PMC9419714

[ref70] Zhu W. Liu J. Lou H. Mu F. Li B. (2024). Influence of smartphone addiction on sleep quality of college students: the regulatory effect of physical exercise behavior. PLoS One 19:e0307162. doi: 10.1371/journal.pone.0307162, PMID: 39058670 PMC11280214

[ref71] Zhu X. Zheng T. Ding L. Zhang X. Li Z. Jiang H. (2023). Exploring associations between social media addiction, social media fatigue, fear of missing out and sleep quality among university students: a cross-section study. PLoS One 18:e0292429. doi: 10.1371/journal.pone.0292429, PMID: 37796805 PMC10553250

